# Supplementary material for: Particle number-based trophic transfer of gold nanomaterials in an aquatic food chain
Source: Nat Commun. 2021 Feb 9;12:899. doi: 10.1038/s41467-021-21164-w (PMC7873305; doi:10.1038/s41467-021-21164-w)
Supplement: Supplementary file 1 — Supplementary Information [file 41467_2021_21164_MOESM1_ESM.pdf]

# Supplementary Information
# Particle number-based trophic transfer of gold nanomaterials in an aquatic food chain

Fazel Abdolahpur Monikh[1,2*], Latifeh Chupani [3], Daniel Arenas-Logo [4], Zhiling Guob [5], Peng Zhang [5], Gopala Krishna Darbha [6], Eugenia Valsami-Jones [5], Iseult Lynch [5], Martina G. Vijver [1], Peter M. van Bodegom [1], Willie J.G.M. Peijnenburg [1,7]

[1] Institute of Environmental Sciences (CML), Leiden University, P.O. Box 9518, 2300 RA Leiden, Netherlands

[2] Department of Environmental & Biological Sciences, University of Eastern Finland, P.O. Box 111, FI-80101 Joensuu, Finland.

[3] South Bohemian Research Center of Aquaculture and Biodiversity of Hydrocenoses, Faculty of Fisheries and Protection of Waters, University of South Bohemia in Ceske Budejovice, Zátiší 728/II, 389 25 Vodňany, Czech Republic

[4] Department of Plant Biology and Soil Science, University of Vigo, As Lagoas, Marcosende, 36310, Vigo, Spain

[5] School of Geography, Earth and Environmental Sciences, University of Birmingham, Edgbaston, Birmingham, B15 2TT, UK

[6] Environmental Nanoscience Laboratory, Department of Earth Sciences, Indian Institute of Science Education and Research Kolkata, Kolkata, Mohanmur, West Bengal, India- 741246

[7] National Institute of Public Health and the Environment (RIVM), Center for Safety of Substances and Products, Bilthoven, Netherlands

* Corresponding Author

Email: f.a.monikh@cml.leidenuniv.nl

Address: Van Steenis Building, Einsteinweg 2, 2333 CC Leiden

ORCID: Fazel A. Monikh 0000-0001-9500-5303

Section 1 | Physicochemical characterization of Au-NMs in Milli-Q water

An extensive characterization of the physicochemical properties of Au-NMs was performed in this study. Transmission electron microscopy (TEM) images of Au-NMs dispersions in Milli-Q (MQ) water were obtained using a JEOL 1400 TEM operated at 80 kV accelerating voltage. About 100 µL of the dispersion of the NMs were pipetted onto copper grids. The grids were kept in darkness at room temperature for 24 h allowing the samples to dry. Figure 1 shows the shape and size distribution of the different Au-NMs used in this study. We must mention that agglomeration of Au-NMs due to the sample preparation for TEM may take place e.g. during drying [1].

The hydrodynamic size was measured by dynamic light scattering and zeta potential was measured using laser Doppler electrophoresis; both measurements were performed using a Zetasizer Nano device (Malvern Panalytical, Netherlands, and the UK). The TEM measured particle sizes were similar to the sizes reported by the supplier. Results showed that the zeta potential increased slightly when the Au-NMs were incubated in the exposure medium.

Table 1 | Physicochemical characterization of the Au-NMs used in this study.

| Au-NMs | Hydro dynamic size (nm) | Transmission electron microscopy measured size (nm) | | Zeta potential (mV) | | |
|---|---|---|---|---|---|---|
| | | Reported by producer | Measured in this study | Reported by producer | Measured in this study in MQ water | Measured in this study in exposure media |
| Spherical 10 nm | 15 ± 6 | 10 | 10 ± 2 | −20 | −25 ± 4 | −19 ± 2 |
| Spherical 60 nm | 71 ± 12 | 60 | 60 ± 5 | −20 | −23 ± 2 | −17 ± 1 |
| Spherical 100 nm | 107 ± 9 | 100 | 100 ± 4 | −20 | −21 ± 3 | −18 ± 4 |
| Rod-shaped 10 × 45 nm | 23 ± 8 | 10 × 45 | 10 ± 2 × 47 ± 3 | −22 | −23 ± 1 | −18 ± 2 |
| Rod-shaped 50 × 100 nm | 74 ± 14 | 50 × 100 | 65 ± 6 × 220 ± 31 | −22 | −21 ± 2 | −17 ± 3 |

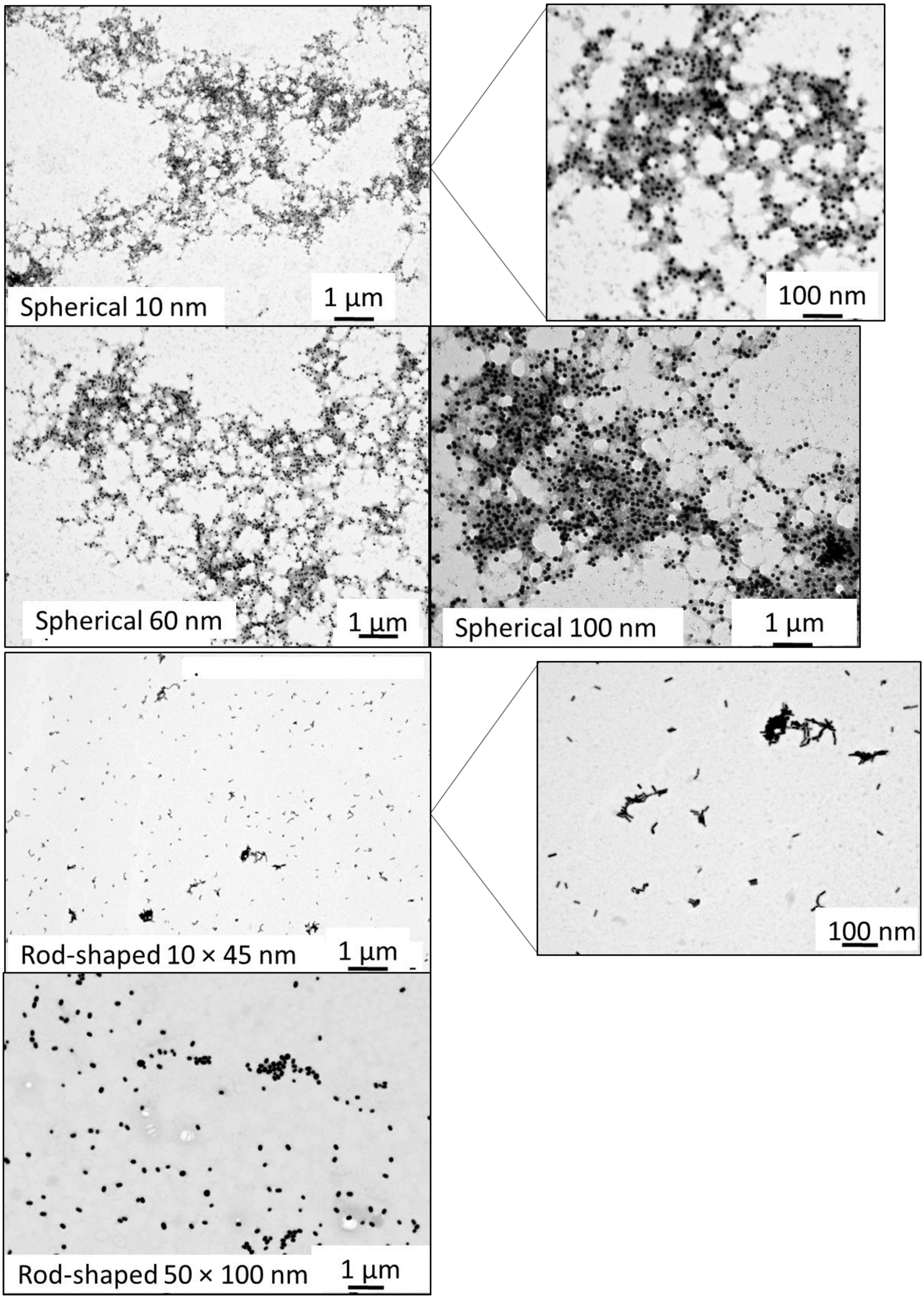


Figure 1 | Transmission electron microscopy images illustrating the shapes of the particles in Milli-Q (MQ) water dispersion. The experiment was repeated independently for three times.

Section 2 | Stability of Au-NMs against agglomeration and dissolution

The stability of the Au-NMs against dissolution and agglomeration was measured by tracing the particle number and size distribution in the algal exposure medium over 72 h which reflects the duration of algal exposure to the Au-NMs. Figure 2a shows that the dissolution of the Au-NMs was less that 0.2 percent of the total Au after 72 h of incubation. The mode of the size distribution of each Au-NMs was measured and plotted in Figure 2b-f (blue lines). The mode was stable over the 72 h of incubation, indicating that the particles were stable against agglomeration and dissolution, otherwise a significant increase in the mode reflecting NM agglomeration or a significant decrease in the mode reflecting dissolution of the NMs would be apparent. The equal number of NMs (Figure 2b-f (black lines) over time further confirms the stability of the particles against agglomeration and dissolution. This findings are in agreement with our previous study [2].

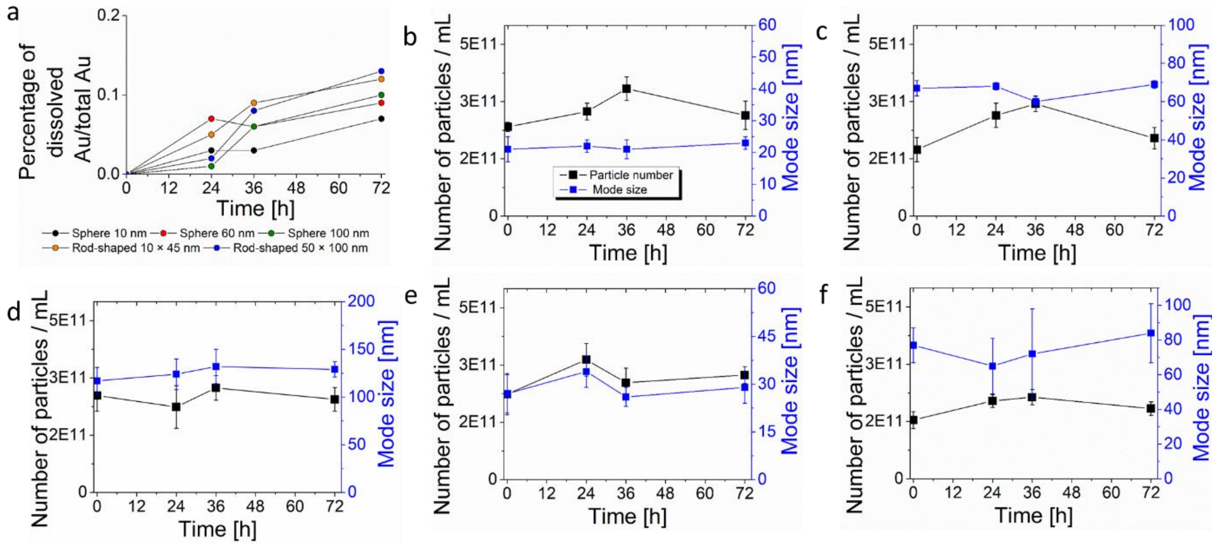


Figure 2 | Stability of the NMs in algal exposure medium over 72 h: a) Dissolution profile of Au-NMs in algal culture medium measured using single particle inductively coupled plasma mass spectrometry (spICP-MS). Data show that dissolution was not significant ($P < 0.05$) over the exposure duration (< 0.2% of total Au). Data were analyzed using one-way ANOVA with Duncan's post hoc test ($p < 0.05$). Agglomeration of the NMs is shown as particle number concentration (black) and mode size (blue) of (b) spherical 10 nm, (c) spherical 60 nm, (d) spherical 100 nm, (e) rod-shaped 10 × 45 nm, (f) rod-shaped 50 × 100 nm Au-NMs in the algal exposure medium without algae. Error bars = Standard Deviation (n=10), independent samples (b-f).

Section 3| Calculation of the Au-NM number

In this study, the Au-NMs are assumed to have a density of 19.3 g $cm^{-3}$. The number of particles in the algal exposure medium was measured using spICP-MS and calculated as follows:

Volume of one Au-NM ($cm^3$) = $V_{single\ Au-NM}(cm^3)$
Volume of total Au-NMs = $V_{total\ Au-NMs}\ (cm^3)$
M = mass
P = density of gold = 19.3 g/$cm^3$
N = number of the Au-NMs

Spherical Au-NMs:

$$\mathrm{V_{single\ Au-NM}}(cm^3) = \frac{4}{3}\pi(r \times 10^{-7})^3 \tag{1}$$

Rod-shaped Au-NMs:

$$\mathrm{V_{single\ Au-NM}}(cm^3) = h\pi(r \times 10^{-7})^2 \tag{2}$$

Particle number

$$\mathrm{V_{total\ Au-NMs}}\ (cm^3) = M/\rho \tag{3}$$

$$\mathrm{N} = \frac{V_{\mathrm{total\ Au-NMs}}\ (cm^3)}{V_{\mathrm{single\ Au-NM}}(cm^3)} \tag{4}$$

Table 2 | Calculation of the mass of Au-NMs at a fixed particle number to ensure that all exposures were equivalent, allowing assessment of particle number at each tropic level.

| Au-NM | Mass (g $mL^{-1}$) | Number $mL^{-1}$ |
|---|---|---|
| Spherical 10 nm | $2.97 \times 10^{-6}$ | $2.93 \times 10^{11}$ |
| Spherical 60 nm | 0.00064 | $2.93 \times 10^{11}$ |
| Spherical 100 nm | 0.002965 | $2.93 \times 10^{11}$ |
| Rod-shaped 10 × 45 nm | $2 \times 10^{-5}$ | $2.93 \times 10^{11}$ |
| Rod-shaped 50 × 100 nm | 0.00111 | $2.93 \times 10^{11}$ |

Section 4 | Total chlorophyll content of the exposed algae

It was reported that Au-NMs have very low toxicity to algae[2]. We investigated the toxicity of the 2.93 × $10^{11}$ Au-NMs of different sizes and shapes to algae (*Pseudokirchinella subcapitata*) (Figure 3a) by measuring the chlorophyll content of the algae using a UV-Vis Spectrophotometer via the absorbance at 670-750 nm. The plasmon resonance of Au-NMs in the exposure media was tested and the results showed (Table 3) that the plasmon resonance does not interfere with the chlorophyll measurement [2]. The toxicity of the exposed cells was calculated with respect to untreated cells. Different algal concentrations of 1000, 5000, 7000 and 10000 cells/mL were measured by an Aquafluor Meter,[2] following absorbance measurement using the UV-vis spectrophotometer. The absorbance value was used to obtain the calibration curve of chlorophyll density. The calibration curve was used to measure the concentration of the chlorophyll in the algae after exposure to the Au-NMs of different sizes and shapes (Figure 3b). The results showed that there was no influence on the concentration of the algal chlorophyll due to the exposure to the Au-NMs compared to the control.

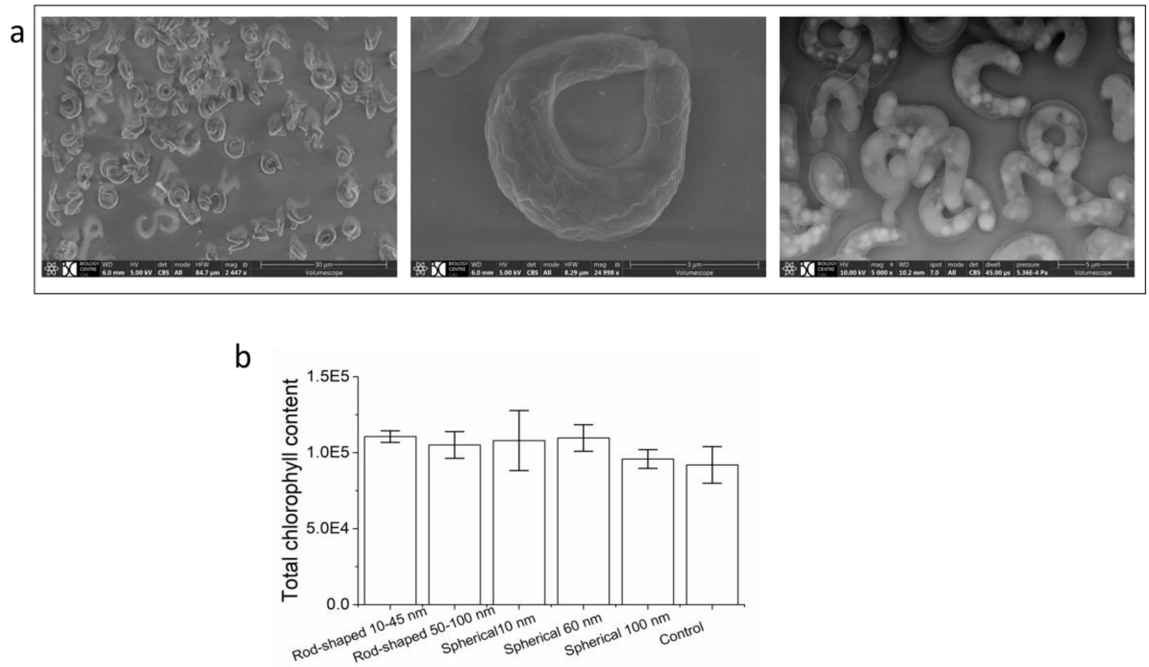


Figure 3 | a) The scanning electron microscope images of the algal cells (*P. subcapitata*). b) Measured total chlorophyll content of algae exposed to Au-NMs of different sizes and shapes. There was no significant difference between NM-treated algae and untreated algae control. Error bars = Standard Deviation (n=15), biological independent samples. The significance differences were analyzed using *t-test* ($p < 0.05$).

Table 3 | The light absorbance of the Au-NMs dispersion measured using a UV-Vis Spectrophotometer

| Au-NMs | Absorbance (nm) |
|---|---|
| Spherical 10 nm | 540 |
| Spherical 60 nm | 560 |
| Spherical 100 nm | 580 |
| Rod-shaped 10 × 45 nm | 640 |
| Rod-shaped 50 × 100 nm | 640 |

Section 5| Removal of loosely attached and unbound Au-NMs from the algae

After incubation, the algal cells were kept at 4 $^0$C to settle down for 48 h. After the sedimentation, the supernatant which contained the unbound Au-NMs was removed. The pellets were collected, dispersed in 10 mL of phosphate buffer saline (PBS, 0.1 M pH 7.5) for 20 minutes and centrifuged (2000 ×g) for 10 min at 4 $^o$C as illustrated in Figure 4. The latter process was repeated three times. The supernatant, which contains the unbound or loosely attached Au-NMs, was removed. The final pellets were weighed and the biomass was recorded based on wet weight (w.w). The pellet was diluted with an algal exposure medium and the concentration of the Au/algal cell was measured using single-cell (sc)ICP-MS.

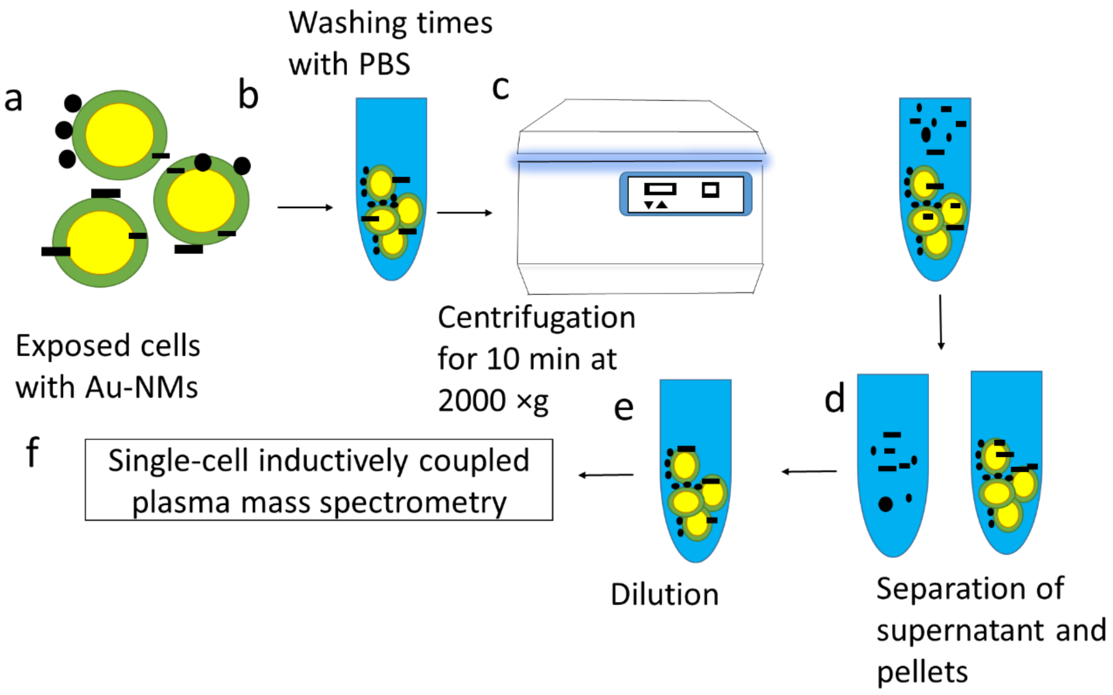


Figure 4 | Visualization of algal cell washing. A-g) The figure illustrates the washing process of the cells using PBS to remove the NMs loosely attached to the cells and to quantify the NMs associated with algae using scICP-MS. After cell exposure to Au-NMs of different sizes and shapes (a), the algae were kept at 4 °C for 48 hours to allow sedimentation of the algae. After sedimentation, the pellet was separated from the supernatant and dispersed in PBS (b) and centrifuged (c). After centrifugation, the supernatant was removed (d) and the pellet was diluted with the algal exposure medium (e) and the concentration of the Au per algal cell was measured using scICP-MS (f).

Section 6| Calculated mass balance

We calculated the mass balance by obtaining the total amount of the Au in the exposure medium, each fraction of the food chain and in the depuration media (Table 4), measured using ICP-MS.

Table 4 | The measured mass of the added Au-NMs in the exposure media, depuration media and different fractions of the food chain to calculate the mass balance

| Au-NMs | Au mass in the algal exposure medium at 0 h (µg $ml^{-1}$) | Au mass in the algal exposure medium after 72 h (µg $ml^{-1}$) | Au mass in the total algal after 72 h (µg $mg^{-1}$ w.w) | Au mass in the daphnids after exposure (µg $mg^{-1}$ w.w) | Au mass in the daphnids culture medium after depuration (µg $L^{-1}$) | Au mass in the total body of fish after exposure (µg/mg w.w) | Au mass in the fish culture medium after depuration (µg $L^{-1}$) |
|---|---|---|---|---|---|---|---|
| Spherical 10 nm | 3.5 ± 0.5 | 0.7 ± 0.04 | 0.0061 ± 0.0004 | $6.7 \times 10^{-6} \pm 7 \times 10^{-7}$ | $9.9 \times 10^{-6} \pm 1.8 \times 10^{-6}$ | $2 \times 10^{-7} \pm 1 \times 10^{-8}$ | 0.003 ± 0.0005 |
| Spherical 60 nm | 612 ± 84 | 345 ± 52 | 0.5224 ± 0.03 | $3.8 \times 10^{-4} \pm 3 \times 10^{-5}$ | $8.2 \times 10^{-4} \pm 9.7 \times 10^{-5}$ | $4 \times 10^{-5} \pm 2 \times 10^{-6}$ | 0.12 ± 0.03 |
| Spherical 100 nm | 2543 ± 392 | 1108 ± 271 | 0.47 ± 0.05 | $6.7 \times 10^{-4} \pm 5.3 \times 10^{-5}$ | $8 \times 10^{-4} \pm 1.1 \times 10^{-5}$ | $1.5 \times 10^{-4} \pm 6 \times 10^{-5}$ | 0.21 ± 0.03 |
| Rod-shaped 10 × 45 nm | 17 ± 3 | 2 ± 0.01 | 0.02 ± 0.006 | $3.4 \times 10^{-5} \pm 5.2 \times 10^{-6}$ | $3.7 \times 10^{-5} \pm 5 \times 10^{-6}$ | $1.5 \times 10^{-6} \pm 3 \times 10^{-7}$ | 0.017 ± 0.004 |
| Rod-shaped 50 ×100 nm | 1207 ± 328 | 765 ± 74 | 0.1923 ± 0.006 | $2.6 \times 10^{-4} \pm 6.4 \times 10^{-5}$ | $3.3 \times 10^{-4} \pm 6.0 \times 10^{-5}$ | $5.6 \times 10^{-5} \pm 3 \times 10^{-6}$ | 0.05 ± 0.007 |

Section 7 |Size distribution of the Au-NMs at each trophic level.

The number-based size distribution of the Au-NMs in all the tested organisms was measured using spICP-MS after particle extraction (see the main text Materials and Methods). The applied method for measurement and NM extraction was validated (in-house). The obtained size distribution of the Au-NMs was plotted and the mode of the size distribution ($M_{size}$) was shown using a red line (Figure 5). The size distribution of the spherical Au-NMs at each trophic level was illustrated in Figure 5a. The $M_{size}$ of the 10 nm spherical Au-NMs in daphnids shifted toward a higher particle size compared to $M_{size}$ in algae. While the $M_{size}$ of the spherical 60 nm and spherical 100 nm shifted toward smaller particle sizes, indicating particle dissolution. The $M_{size}$ of the Au-NMs in the fish intestine was similar to $M_{size}$ determined in daphnids while the $M_{size}$ in the fish brain was larger. The size distribution of the rod-shaped Au-NMs at each trophic level was illustrated in Figure 5b. The $M_{size}$ of the rod-shaped 10 × 45 nm Au-NMs in daphnids shifted toward a higher particle size compared to $M_{size}$ in algae, indicating agglomeration of the NMs. The $M_{size}$ of the rod-shaped 50 × 100 nm Au-NMs shifted toward smaller particle sizes, indicating particle dissolution. The $M_{size}$ of the Au-NMs in the fish intestine was similar to $M_{size}$ determined in daphnids while the $M_{size}$ in the fish brain and liver was larger.

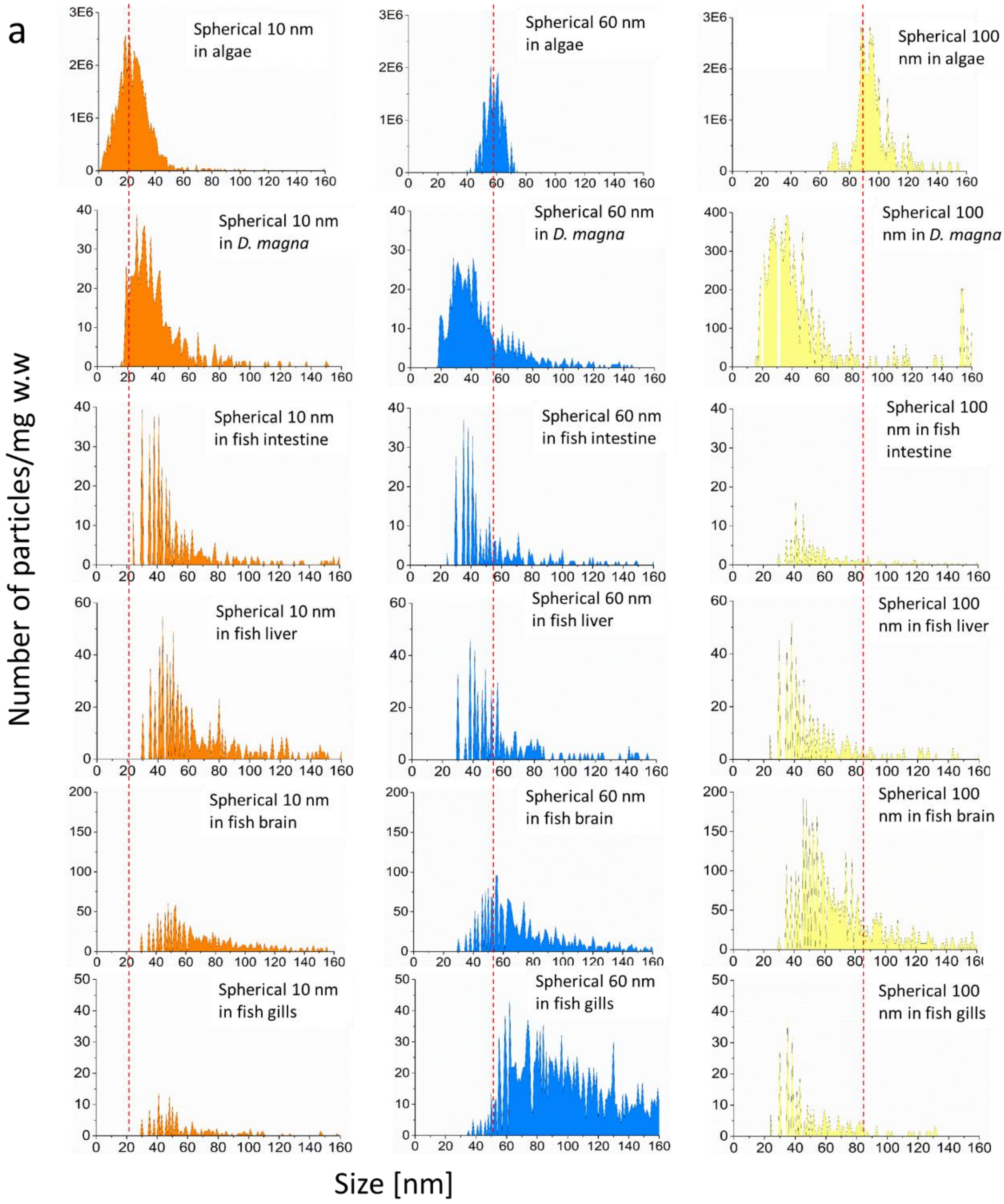
a
Number of particles/mg w.w
Size [nm]
Spherical 10 nm in algae
Spherical 60 nm in algae
Spherical 100 nm in algae
Spherical 10 nm in D. magna
Spherical 60 nm in D. magna
Spherical 100 nm in D. magna
Spherical 10 nm in fish intestine
Spherical 60 nm in fish intestine
Spherical 100 nm in fish intestine
Spherical 10 nm in fish liver
Spherical 60 nm in fish liver
Spherical 100 nm in fish liver
Spherical 10 nm in fish brain
Spherical 60 nm in fish brain
Spherical 100 nm in fish brain
Spherical 10 nm in fish gills
Spherical 60 nm in fish gills
Spherical 100 nm in fish gills

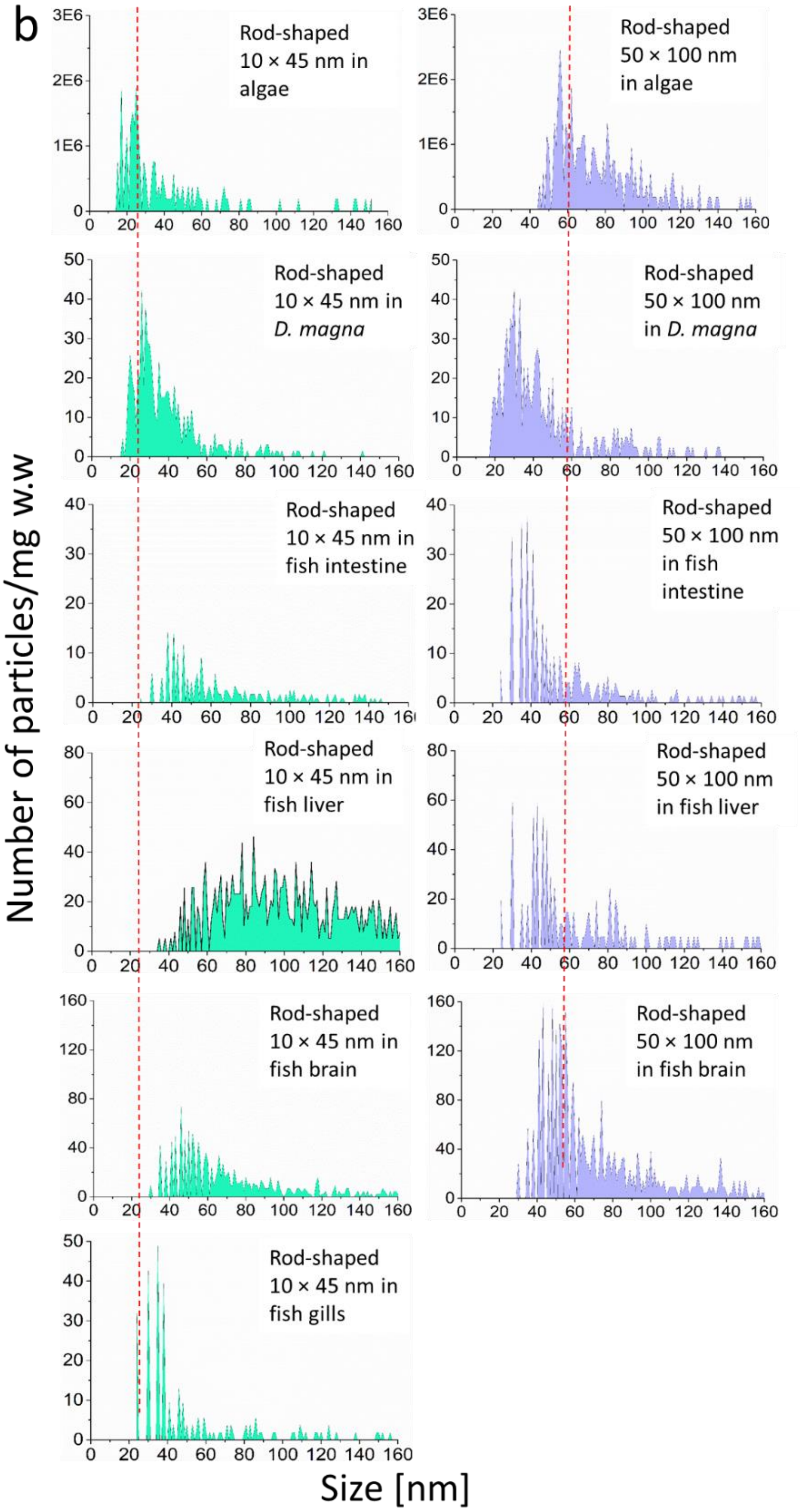


Figure 5 | Size distribution and mode size ($M_{size}$) of Au-NMs at each trophic level. The red dashed line in each graph shows the $M_{size}$ of the Au-NMs size distribution at the first trophic level (algae) and is used as a baseline to monitor shifts in $M_{size}$ of particles at higher trophic levels. Trophic shifts in NM size are evident in daphnids and fish tissues for spherical Au-NMs (a) and rod-shaped NMs (b).

Section 8| Dissolution of Au-NMs in fish plasma

Because obtaining enough plasma from zebrafish is problematic due to their small size which also leads to sacrificing a high number of fish, some healthy adult carp fish were selected regardless of the gender and kept in the lab for acclimatization. The fish plasma was derived from the carp blood with a total protein concentration of 45 g $L^{-1}$. The plasma was kept at -80 °C and used for all experiments. To perform the dissolution test in the fish plasma, aliquots of Au-NMs of different size and shapes (spherical 10 nm, spherical 60 nm, spherical 100 nm, rod-shaped 10 × 45 nm and rod-shaped 50 × 100 nm Au-NMs) were dispersed in PBS (pH 7.5) to reach a final concentration of 20 mg $L^{-1}$ of the Au-NMs. The dispersions were sonicated for 5 min using a bath sonicator (35 kHz frequency, DT 255, Bandelin electronic, Sonorex digital, Berlin, Germany) in an ice bath for 5 min. After sonication, aliquots of the Au-NM dispersions were incubated in 1 mL of fish plasma (10 times diluted; containing 4.5 g $L^{-1}$ proteins) to reach a final concentration of 10 mg $L^{-1}$ of Au-NMs. After 24 h of incubation in the plasma, the samples were digested using 5% Tetramethylammonium hydroxide (TMAH) for 1 h to digest the biomolecules and free the Au ions sorbed to these molecules if such adsorption took place after the release of Au ions from the particles. The obtained samples were measured using spICP-MS to differentiate between Au ions and particles.

Section 9| Mass-based biomagnification factor

The mass-based biomagnification factor (MBMF) of the Au-NMs in fish for each tissue was calculated separately to determine the difference between each tissue (Figure 6). The MBMFs were calculated by dividing the total mass of each Au-NM in fish tissue to that in algae. The MBMF of Au-NMs in the fish brain was higher than other tissues, except for the rod-shaped 10 × 45 nm Au-NMs which was higher in the liver. This demonstrates that the fish brain followed by the liver are the potential targets for Au accumulation upon fish exposure to feed containing Au-NMs, regardless of NM size and shape.

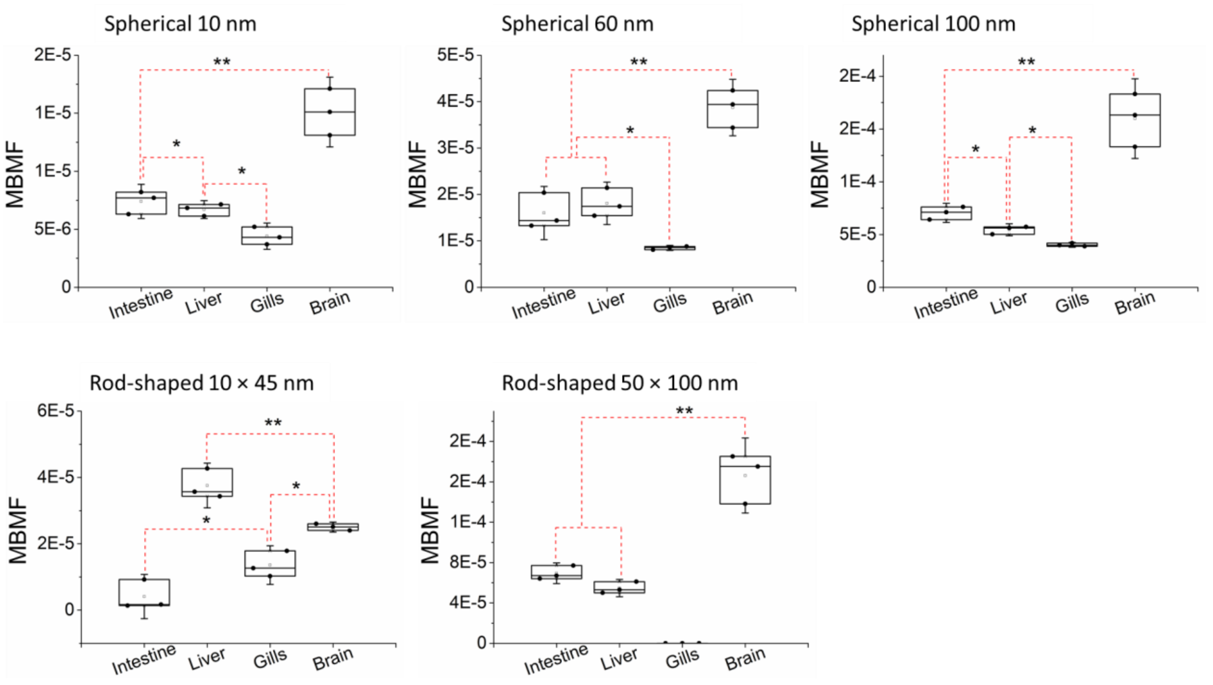


Figure 6 | Mass-based biomagnification factor (MBMF) calculated for Au-NMs in fish. The MBMFs of spherical 10, 60 and 100 nm Au-NMs and rod-shaped 10 × 45 nm and 50 × 100 nm Au-NMs were calculated by dividing the total mass of the Au in each fish tissue to the total mass of Au in algae. Data (n=3) were analyzed using one-way ANOVA followed by Duncan's post hoc test (b) ($p < 0.001$; $p < 0.05$). Box plots (b) indicate median (middle line), 25th, 75th percentile (box) and standard deviation (whiskers).

Section 10| Measuring Au-NMs dissolution and agglomeration in algal culture medium using spICP-MS

Aliquots of the Au-NMs dispersions were taken immediately after sonication and put in the algal culture medium without cells to reach a final concentration of 1 mg $L^{-1}$ of the NMs. About 1 mL of the samples were taken to measure the total Au in the medium. The samples were immediately put on a shaker at 22 °C to mimic the algal culture conditions. To separate dissolved and particulate Au, aliquots of the dispersions were taken at each time point (0, 24, 36 and 72 h) and measured using spICP-MS with the instrument settings as shown in Table 5. The calibration curve of the Au measured using ICP-MS is reported in Figure 7. Changes in the concentrations of the dissolved Au in the samples over time were determined to examine the dissolution during the 72 h of exposure [1]. The number of particles and the size distribution were measured using spICP-MS. An increase in the concentration of the Au ions in each treatment suggests NM dissolution and a decrease in the NM number suggest NM agglomeration (or dissolution and re-precipitation into a few larger particles). Sample preparation for spICP-MS was carried out following the method reported by Abdolahpur Monikh et al [1].

Table 5 | Single-particle inductively coupled plasma mass spectrometry settings

| Single-particle ICP-MS parameters | |
|---|---|
| Radio frequency power | 1600 W |
| Nebulizer type | Quartz nebulizer for NexIONs 300 |
| Spray chamber type | Glass cyclonic |
| Plasma gas flow | 18 L $min^{-1}$ |
| Nebulizer gas flow | 1.2 L $min^{-1}$ |
| Auxiliary gas flow | 1.12 L $min^{-1}$ |
| Isotopes | $^{197}Au$ |
| Dwell time | 50 µs |
| Acquisition time | 100 s |

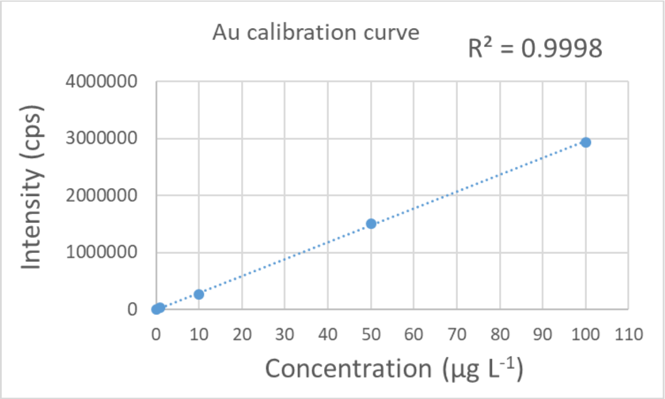


Figure 7 | Calibration cure of Au measured by inductively coupled plasma mass spectrometry.

Section 11| Algal culturing and exposure

The unicellular algae *P. subcapitata* was cultured as follows. Algae, 5 × $10^3$ cells/mL in accordance with the OECD recommended guideline (OECD 201), were kept in algal medium Woods Hole for exposure testing at the laboratory of Leiden University, the Netherlands. The cells were exposed to 2.94 × $10^{11}$ particles/mL of different NM sizes or shapes during their steady-state phase of growth (after 6-7 days) for 72 h. Along with the samples, we ran several control treatments including algae without any exposure. The flasks containing the exposed algae were placed in a climate chamber (22 $^0$C) at a light intensity of 70 mE $m^{-2}$ $s^{-1}$. The flasks containing algae and the NMs as well as the controls were constantly shaken at 80 rpm using a G10 Gyratory Shaker (Washington, the US) to prevent sedimentation of the algae. After exposure, the cells were kept at 4 $^0$C for 48 h to allow sedimentation. The pellets of the algal cells were separated from the supernatant, which was discarded, and used for further analysis.

Section 12| Single cell ICP-MS parameters

The mass of the Au-NMs in the algae cells was measured on a cell-by-cell basis using scICP-MS. The sample introduction was accomplished with the Single Cell Micro DX Autosampler, the high-efficiency nebulizer (HEN), and the Asperon spray chamber. In scICP-MS, the autosampler utilizes a syringe pump for accurate, low-flow delivery of the cells to the HEN, which aspirates the cell suspensions. This combination of autosampler and HEN is critical to ensuring that the cells do not burst when they are nebulized. The Asperon spray chamber provides a laminar flow for maximum delivery of the cells to the plasma; for maximum efficiency it is a full consumption nebulizer meaning that all of the samples are delivered to the plasma, thus minimizing loss of sample. All data acquisition was accomplished with the Syngistix Single-Cell Application Module. The instrument settings used are shown in Table 6.

Table 6 | Single-cell ICP-MS parameters

| | |
|---|---|
| Sample uptake rate | 0.02 mL $min^{-1}$ |
| Nebulizer | MEINHARD HEN (High Efficiency Quartz Concentric Nebulizers) |
| Spray chamber | Asperon |
| Injector | 2.0 mm id Quartz |
| RF power | 1600 W |
| Dwell time | 50 µs |
| Acquisition time | 40 s |
| Replicates per sample | 1 |
| Transport efficiency | 41% |

Section 13| Au-NM extraction method

The sample preparation followed the generic scheme suggested by Abdolahpur Monikh et al.[3] for extracting NMs from biological tissue. Briefly, the organisms (algae, daphnids and fish tissues) were homogenized separately using a T 10 basic ULTRA-TURRAX® homogenizer (IKA, Staufen, Germany) with a stator diameter of 8 mm, rotor diameter of 6.1 mm, and the maximum circumferential speed of 9.6 m/s. The homogenized tissues were diluted with 1 mL of MQ water and sonicated using a model P30H Elmasonic bath sonicator (Elma Schmidbauer, Singen, Germany) for 10 min to aid in breaking down tissue. The resulting samples were digested using 5% TMAH. The duration of the sample digestion was 1 h. The obtained suspensions were dispersed using 5 mL of 0.05% sodium dodecyl sulfate solution, followed by sonication for 5 min at a delivered power of 40 W. We adjusted the pH of the dispersion to 8–8.5 using NaOH.

Section 14| In-house validation of the NM extraction method

The preparation process may influence the NMs and lead to particle agglomeration and/or dissolution [1]. To evaluate the influence of the NM extraction method on the NMs, spike recovery experiments were conducted by the addition of 1 mg $Kg^{-1}$ of the Au-NMs into MQ water. The size distribution of the Au-NMs in MQ water was measured. The Au-NMs were extracted from the MQ water using the method developed for NM extraction. Possible alterations in the particle size distribution of the NMs due to the sample preparation method were evaluated by comparing the size distribution of the pristine NMs before the sample preparation process with the size distribution of the NMs after sample preparation. The results (Figure 8) show that the NM extraction method did not influence the size distribution of the NMs.

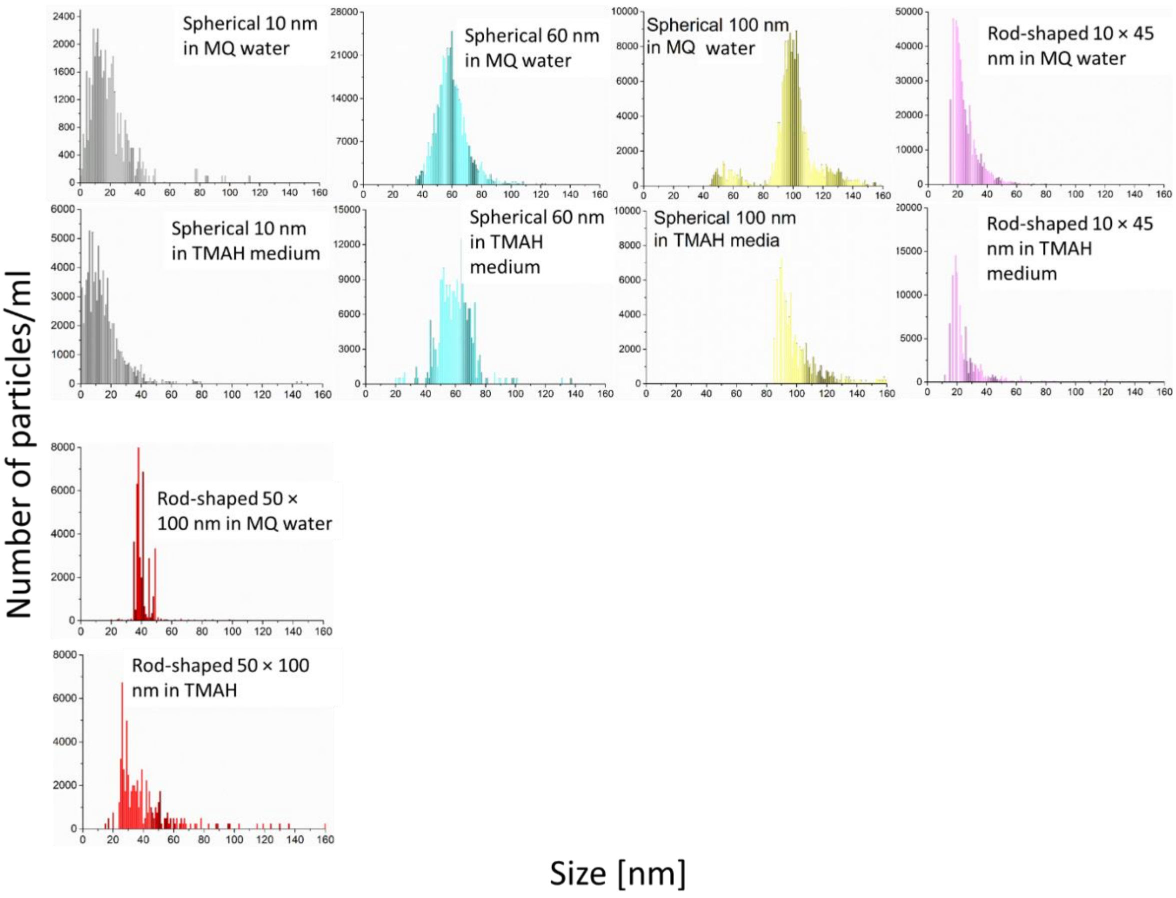


Figure 8 | In-house validation of the NM extraction method. Evaluation of the influence of the NM extraction method on the size distribution of the Au-NMs. The figure shows the size distribution of the Au-NMs as measured by spICP-MS in MQ water before and after extraction.

References


1. Abdolahpur Monikh F, Chupani L, Vijver MG, Vancova M, Peijnenburg WJGM. Analytical approaches for characterizing and quantifying engineered nanoparticles in biological matrices from an (eco)toxicological perspective: old challenges, new methods and techniques. *Sci Total Environ* **660**, 1283-1293 (2019).

2. Abdolahpur Monikh F*, et al.* Do the joint effects of size, shape and ecocorona influence the attachment and physical eco(cyto)toxicity of nanoparticles to algae? *Nanotoxicology*, 1-16 (2019).

3. Abdolahpur Monikh F*, et al.* Method for Extraction and Quantification of Metal-Based Nanoparticles in Biological Media: Number-Based Biodistribution and Bioconcentration. *Environ Sci Technol* **53**, 946-953 (2019).
