# Supplementary material for: Particle number-based trophic transfer of gold nanomaterials in an aquatic food chain
Source: Nat Commun. 2021 Feb 9;12:899. doi: 10.1038/s41467-021-21164-w (PMC7873305; doi:10.1038/s41467-021-21164-w)
Supplement: Supplementary file 3 — Reporting Summary [file 41467_2021_21164_MOESM3_ESM.pdf]

nature research

Corresponding author(s): Fazel Abdolahpur Monikh

Last updated by author(s): Dec 5, 2020

Reporting Summary

Nature Research wishes to improve the reproducibility of the work that we publish. This form provides structure for consistency and transparency in reporting. For further information on Nature Research policies, see our Editorial Policies and the Editorial Policy Checklist.

Statistics

For all statistical analyses, confirm that the following items are present in the figure legend, table legend, main text, or Methods section.

n/a	Confirmed	
[ ]	[x]	The exact sample size (*n*) for each experimental group/condition, given as a discrete number and unit of measurement
[ ]	[x]	A statement on whether measurements were taken from distinct samples or whether the same sample was measured repeatedly
[ ]	[x]	The statistical test(s) used AND whether they are one- or two-sided<br>*Only common tests should be described solely by name; describe more complex techniques in the Methods section.*
[x]	[ ]	A description of all covariates tested
[ ]	[x]	A description of any assumptions or corrections, such as tests of normality and adjustment for multiple comparisons
[ ]	[x]	A full description of the statistical parameters including central tendency (e.g. means) or other basic estimates (e.g. regression coefficient) AND variation (e.g. standard deviation) or associated estimates of uncertainty (e.g. confidence intervals)
[ ]	[x]	For null hypothesis testing, the test statistic (e.g. *F*, *t*, *r*) with confidence intervals, effect sizes, degrees of freedom and *P* value noted<br>*Give P values as exact values whenever suitable.*
[x]	[ ]	For Bayesian analysis, information on the choice of priors and Markov chain Monte Carlo settings
[x]	[ ]	For hierarchical and complex designs, identification of the appropriate level for tests and full reporting of outcomes
[x]	[ ]	Estimates of effect sizes (e.g. Cohen's *d*, Pearson's *r*), indicating how they were calculated

Our web collection on statistics for biologists contains articles on many of the points above.

Software and code

Policy information about availability of computer code

Data collection	No software was used
Data analysis	SPSS 23 and OriginLab 9.1

For manuscripts utilizing custom algorithms or software that are central to the research but not yet described in published literature, software must be made available to editors and reviewers. We strongly encourage code deposition in a community repository (e.g. GitHub). See the Nature Research guidelines for submitting code & software for further information.

Data

Policy information about availability of data

All manuscripts must include a data availability statement. This statement should provide the following information, where applicable:

- Accession codes, unique identifiers, or web links for publicly available datasets
- A list of figures that have associated raw data
- A description of any restrictions on data availability

The authors declare that the data supporting the findings of this study are available within the article and its supplementary information files.

Field-specific reporting

Ecological, evolutionary & environmental sciences study design

All studies must disclose on these points even when the disclosure is negative.

Study description	The trophic transfer of gold nanomaterials (Au-NMs) in an assembled aquatic food chain (algae-daphnids-fish) was investigated. The Au-Ms were dispersed in Milli-Q (MQ) water and sonicated using a tip sonicator. We used 5 different types of Au-NMs. Each type of the Au-NMs was used to expose the organisms separately and a group was used as a control group (6 groups in total). To understand the influence of the NMs on algae, we divided the algae (Pseudokirchinella subcapitata,) into 6 groups (15 replicate per group). The algae were used to feed the daphnids. Since we required the statistical power of the experiment to be high while minimizing the experiment with vertebrate, we chose 54 zebrafish (9 per group). Adult zebrafish should be fed with almost 1% of their body weight, then we chose the number of daphnids to be 10, which is almost equal to 100 mg w.w. These led to the sample choice of 90 daphnids per treatment.<br>Fish were fed with exposed daphnids (10 daphnids, which were equal to almost 100 mg w.w., per day) for 21 days. Exposures of zebrafish were conducted in 30 L glass aquaria and water was changed every 36 h. Three replicates were used for each treatment and three fish were used in each replicate. After exposure, the fish were held for 48 h without feeding to empty their stomachs and the concetation of the total Au in the medium was measured to obtain the Au depuration by fish. The intestine, liver, gills, and brains of each fish were immediately dissected, weighed, and digested for NM extraction. The extracted NMs were prepared for spICP-MS analysis to measure the number of the particles in each tissue, the particle sizes, and the fraction (if any) of dissolved ions.
Research sample	Adult zebrafish (Danio rerio, 3 months old) were maintained for 30 days in the laboratory conditions to become acclimated to the zebrafish culture water (carbon-filtered, dechlorinated tap water). The zebrafish (54 individual of mixed sex) were kept at 22 °C on a 14:10 h light:dark cycle. The number of the zebrafish were selected to obtaine 9 individal fish per treatment. The sample were representative containing equal male and female fish. During acclimatization, zebrafish were fed with unexposed live D. magna (10 adult daphnids of 10 days age, which were equal to almost 100 mg w.w., per day).
Sampling strategy	Fish were fed with NM-exposed daphnids for 21 days. After 21 days the exposure stopped the fish were held for 48 h without feeding to empty their stomachsand. The fish were separated and some of them were killed after Immobilization by submersion in ice water. The fish were dissected to remove the intestine, liver, gills and brain. The tissues were digested using TMAH to extract the NMs w After NMs extraction, the samples were analysed using spICP-MS (h, green arrow).<br>The ecologically most relevant response variable can be measured in living organisms which does not die accidentally. If the animal dies accidentally during the test, the replicate is excluded from the analysis. The number of the samples was selected to have enough data points per treatment (9 fish per treatment) as described in previous ecotoxicological studies (https://doi.org/10.1039/D0EN00240B; https://doi.org/10.1002/etc.2226) while minimizing performing experiment with vertebrates.<br>Data were evaluated statistically for normality using a Kolmogorov–Smirnov test in SPSS version 23.0. One-way analysis of variance (ANOVA), followed by Duncan's post hoc test, was performed to determine statistically significant differences between samples.
Data collection	The purpose of this test is to determine the trophic transfer of NMs from algae to daphnids and from daphnids to fish. The total number of Au-NMs and mass concentration of Au in each organism (algae, daphnids and fish) should be measured for each size and shape of NMs. The concentration of the Au-NMs was measured using single-particle inductively coupled plasma mass spectrometry (ICP-MS) and the concentration of Au was measured using ICP-MS after acid digestion of the samples. Performing the exposure experiment was carried out by F.A.M and L.C. The fish plasma was extracted from fish by L.C. The exposure of Au-NMs to fish plasma was carried out by F.A.M and D.A.L. The single-particle ICP-MS and the ICP-MS measurement was carried out by F.A.M. Characterization of the Au-NMs was performed using a transmission electron microscope and dynamic light scattering by F.A.M and G.K.D.
Timing and spatial scale	The experiment started from:<br>January 2018-May 2018: developing protocol for Au-NMs extraction and method validation at Leiden University, the Netherlands<br>July 2018-June 2018: Performing Au-NMs characterization experiment at Leiden University, the Netherlands<br>July 2018-August 2018: Performing Au-NMs characterization experiment and culturing algae and Daphnis and acclimatizing zebrafish at the University of South Bohemia, Czech Republic<br>July 2018-November 2018: exposure of the organisms to Au-NMs and performing the food chain experiments at the University of South Bohemia, Czech Republic<br>November 2018-March 2019: Performing the particle extraction experiment and particle measurement at the University of Birmingham, the UK.<br>March 2019-August 2019: Performing the particle extraction experiment and Au measurement at Leiden University, the Netherlands.<br>November 2018-December 2019: Data analysis
Data exclusions	No data was excluded
Reproducibility	All attempts to repeat the experiment were successful. The experiment for particle extraction from the organisms and particle analysis using the developed method was replicated for three times.
Randomization	To increase the number of biological independent samples we selected 54 adult fish (27 female and 27 male) and divided them between 6 groups (three replicates for each treatment and 3 fish in each replicate). The organisms were randomly distributed between the groups with a mixture of males and females. We randomly distributed adult daphnids in 6 groups (100 daphnids per group). Each fish was fed with 10 daphnids for each treatment per day because the weight of 10 adult daphnids was almost 100 mg which is suitable as feed for zebrafish. The fish were fed randomly with the exposed daphnids of the same particular treatment.
Blinding	Blinding was not relevant to this study. Here, knowing certain information and treatments by the researchers involved in the research did not influence the study findings. In this experiment, most of the information was generated for the first time. For example, we did not know which size and which shape of the particles may induce or show effect and which effect. We also did not know how

organisms may react to exposure to the nanomaterials.

Did the study involve field work? ☐ Yes ☒ No

Reporting for specific materials, systems and methods

We require information from authors about some types of materials, experimental systems and methods used in many studies. Here, indicate whether each material, system or method listed is relevant to your study. If you are not sure if a list item applies to your research, read the appropriate section before selecting a response.

Materials & experimental systems

n/a	Involved in the study
☒	☐ Antibodies
☒	☐ Eukaryotic cell lines
☒	☐ Palaeontology and archaeology
☐	☒ Animals and other organisms
☒	☐ Human research participants
☒	☐ Clinical data
☒	☐ Dual use research of concern

Methods

n/a	Involved in the study
☒	☐ ChIP-seq
☒	☐ Flow cytometry
☒	☐ MRI-based neuroimaging

Animals and other organisms

Policy information about studies involving animals; ARRIVE guidelines recommended for reporting animal research

Laboratory animals	algae (Pseudokirchinella subcapitata, 6-8 days old)<br>Daphnia Magna: female (10-15 days old)<br>Zebrafish (Danio rerio): male and female (3 months old)
Wild animals	the study did not involve wild animals
Field-collected samples	The study did not involve samples collected from the field.
Ethics oversight	All experiments were performed with the approval of the ethics committee of the Research Center of Aquaculture and Biodiversity of Hydrocenoses, the University of South Bohemia in Ceske Budejovice, Czech Republic (MSMT-6744/2018-4).

Note that full information on the approval of the study protocol must also be provided in the manuscript.
